# Complete chloroplast genome sequence of *Ampelocalamus scandens* (Arundinarodae)

**DOI:** 10.1080/23802359.2020.1768939

**Published:** 2020-06-01

**Authors:** Lili Fan, Yangyang Zhang, Muhammad Waqqas Khan Tarin, Tianyou He, Jundong Rong, Yushan Zheng

**Affiliations:** a College of Forestry, Fujian Agriculture and Forestry University, Fuzhou, PR China; b College of Landscape, Fujian Agriculture and Forestry University, Fuzhou, PR China

**Keywords:** *Ampelocalamus scandens*, plastid genome, phylogeny, Arundinarodae

## Abstract

*Ampelocalamus scandens* is native to Guizhou Province, China, and grows at an altitude of 260–320 m. It can be used as a raw material for weaving and papermaking. In the current study, the complete chloroplast (cp) genome of *A. scandens* was sequenced and is reported for the first time. The complete cp sequence was 139,504 bp, include large single-copy (LSC), small single-copy (SSC), and a pair of invert repeats (IR) region of 83,103 bp, 12,813 bp, and 21,793 bp, respectively. Besides, the plastid genome comprised a total of 132 genes, including protein-coding, tRNA, and rRNA genes as 85, 39, and 8 genes, respectively. Phylogenetic analysis based on 28 cp genomes reveals that *A. scandens* is closely associated with *Ampelocalamus melicoideus* in Arundinarodae.

*Ampelocalamus scanden* is a vine-like bamboo with a pole length of up to 10 m. It is a unique bamboo species native to Guizhou Province, China (Geng and Wang 1996). *A. scandens* is listed as threatened species in the International Union for Conservation of Nature (IUCN) red list in 2007, with an evaluation of Critically Endangered species. Therefore, there is a need to protect and expand its distribution (Meng et al. 2012). In this study, we reported the complete chloroplast genome (cp) of *A. scandens* based on Illumina pair-end sequencing data. Fresh leaves samples of *A. scandens* were collected from Fujian province, China (Fujian Agriculture and Forestry University, Bamboo Garden, Fuzhou; located at 26°5′7″N, 119°14′16″E, and dried into silica gel instantly. The specimen were kept in the Herbarium of College of Forestry, Fujian Agriculture and Forestry University (code HTY006). DNA was extracted from fresh leaves tissue, with 500 bp randomly interrupted sequence by the Covaris ultrasonic breaker for library construction. The constructed library was sequenced PE150 by Illumina Hiseq Xten platform, with ∼2GB data generated. Illumina data were filtered by a script in the cluster (default parameter: -L5, -p0.5, -N0.1). The complete plastid genome of *Arundinaria faberi* (GeneBank accession: JX513414) as a reference and plastid genome of *A. scandens* were assembled by GetOrganelle pipe-line (https://github.com/Kinggerm/GetOrganelle), which can get the plastid-like reads, and these were viewed and edited by Bandage (Wick et al. 2015). The cp genome annotation was assembled based on the comparison by Geneious v 11.1.5 (Biomatters Ltd, Auckland, New Zealand) (Kearse et al. 2012). The annotation result was drawn with the online tool OGDRAW (http://ogdraw.mpimp-golm.mpg.de/) (Lohse et al. 2013).

The complete plastid genome sequence of *A. scandens* (GenBank accession:MT380008) was 139,504 bp in length, with a large single-copy (LSC) region of 83,103 bp, a small single-copy (SSC) region of 12,813 bp, and a pair of inverted repeat (IR) regions of 21,793 bp. The complete chloroplastid genome contains 132 genes, having 85 protein-coding genes, 39 tRNA genes, and 8 rRNA genes. The complete genome GC contents were 38.9%. To reveal the phylogenetic position of *A. scandens* with other members of Arundinarodae, a phylogenetic analysis was performed based on 22 complete chloroplast genomes of Arundinarodae, and six taxa (*Bambusa bambos, Bambusa emeiensis, Bambusa multiplex, Bambusa ventricosa, Bambusa oldhamii, Bambusa arnhemica*) as outgroups; downloaded from NCBI GenBank. The sequences were aligned by MAFFT v7.307 (Katoh and Standley 2013), and the phylogenetic tree was constructed by RAxML (Stamatakis 2014) The phylogenetic tree showed that *A. scandens* was close to its confamilial plant, *Ampelocalamus melicoideus* (Figure 1).

**Figure 1.**
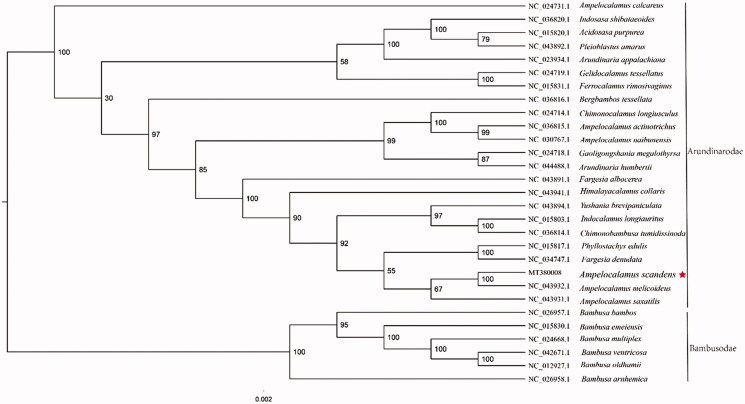
Phylogenetic analysis of 22 species of Arundinarodae and six taxa (*Bambusa bambos, Bambusa emeiensis, Bambusa multiplex, Bambusa ventricosa, Bambusa oldhamii, Bambusa arnhemica*) as outgroup based on plastid genome sequences by RAxML, bootstrap support value near the branch.

## Data Availability

The data that support the findings of this study are openly available in https://www.ncbi.nlm.nih.gov/ GeneBank with following accession number MT380008.

## References

[CIT0001] Geng BJ, Wang ZP. 1996. Compilation of “Volume of Chinese Flora” nine volumes and one volume (Gramineae-Bamboo Subfamily). J Bamboo Res. 9(1):77–79.

[CIT0002] Katoh K, Standley DM. 2013. MAFFT multiple sequence alignment software version 7: improvements in performance and usability. Mol Biol Evol. 30(4):772–780. 23329690 10.1093/molbev/mst010 PMC3603318

[CIT0003] Kearse M, Moir R, Wilson A, Stones-Havas S, Cheung M, Sturrock S, Buxton S, Cooper A, Markowitz S, Duran C, et al. 2012. Geneious basic: an integrated and extendable desktop software platform for the organization and analysis of sequence data. Bioinformatics. 28(12):1647–1649. 22543367 10.1093/bioinformatics/bts199 PMC3371832

[CIT0004] Lohse M, Drechsel O, Kahlau S, Bock R. 2013. OrganellarGenomeDRAW – a suite of tools for generating physical maps of plastid and mitochondrial genomes and visualizing expression data sets. Nucleic Acids Res. 41(Web Server issue):W575–W581. 23609545 10.1093/nar/gkt289 PMC3692101

[CIT0005] Meng WY, Gou GQ, He LJ, et al. 2012. Research on the tissue culture of endemic bamboo in Guizhou. J Mountain Agri Biol. 31(02):116–118.

[CIT0006] Stamatakis A. 2014. RAxML version 8: a tool for phylogenetic analysis and post-analysis of large phylogenies. Bioinformatics. 30(9):1312–1313. 24451623 10.1093/bioinformatics/btu033 PMC3998144

[CIT0007] Wick RR, Schultz MB, Zobel J, Holt KE. 2015. Bandage: interactive visualization of de novo genome assemblies. Bioinformatics. 31(20):3350–3352. 26099265 10.1093/bioinformatics/btv383 PMC4595904

